# High-Temperature Creep Resistance of FeAlOY ODS Ferritic Alloy

**DOI:** 10.3390/ma17204984

**Published:** 2024-10-11

**Authors:** Petr Dymáček, Milan Jarý, Denisa Bártková, Natália Luptáková, Štepán Gamanov, Petr Bořil, Vjaceslav Georgiev, Jiří Svoboda

**Affiliations:** 1 Institute of Physics of Materials CAS, Žižkova 22, 616 00 Brno, Czech Republic; jary@ipm.cz (M.J.); bartkova@ipm.cz (D.B.); luptakova@ipm.cz (N.L.); gamanov@ipm.cz (Š.G.); boril@ipm.cz (P.B.); svobj@ipm.cz (J.S.); 2 Faculty of Mechanical Engineering, Brno University of Technology, Technická 2, 616 00 Brno, Czech Republic; 3 Central European Institute of Technology, Brno University of Technology, Purkyňova 656/123, 612 00 Brno, Czech Republic; 4 Faculty of Electrical Engineering, University of West Bohemia, Univerzitní 8, 306 14 Plzeň, Czech Republic; georg@fel.zcu.cz

**Keywords:** oxide-dispersion-strengthened (ODS) alloy, mechanical alloying, high-temperature, creep, threshold stress

## Abstract

A significant effort in optimizing the chemical composition and powder metallurgical processing led to preparing new-generation ferritic coarse-grained ODS alloys with a high nano-oxide content. The optimization was aimed at high-temperature creep and oxidation resistance at temperatures in the range of 1100–1300 °C. An FeAlOY alloy, with the chemical composition Fe–10Al–4Cr–4Y_2_O_3_ (wt. %), seems as the most promising one. The consolidation of the alloy is preferably conducted by hot rolling in several steps, followed by static recrystallization for 1 h at 1200 °C, which provides a stable coarse-grain microstructure with homogeneous dispersion of nano-oxides. This represents the most cost-effective way of production. Another method of consolidation tested was hot rotary swaging, which also gave promising results. The compression creep testing of the alloy at 1100, 1200, and 1300 °C shows excellent creep performance, which is confirmed by the tensile creep tests at 1100 °C as well. The potential in such a temperature range is the target for possible applications of the FeAlOY for the pull rods of high-temperature testing machines, gas turbine blades, or furnace fan vanes. The key effort now focuses on expanding the production from laboratory samples to larger industrial pieces.

## 1. Introduction

**Figure 1:**
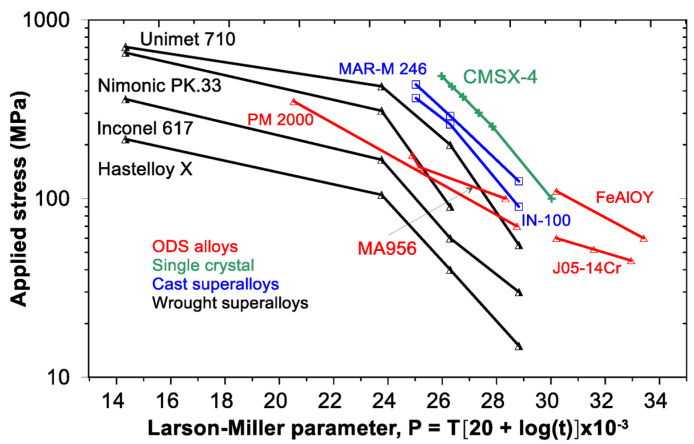
Comparison of creep performance of commercial and experimental alloys [4].

Oxide-dispersion-strengthened (ODS) alloys represent a group of materials with a broad potential for high-temperature applications up to 1300 °C [1,2,3]. They are also intensively studied for nuclear applications, such as fourth-generation fission or fusion reactors, due to low activation by neutrons [4,5]. Commonly, the ODS alloy microstructure consists of the metallic matrix strengthened by dispersion of stable yttrium-based oxides of a typical size between 5 and 30 nm and of a volume fraction of about 0.5%. Depending on the application temperature and oxidation environment, the matrix can be represented by steel or ferritic alloy [2,6,7,8], austenitic alloy, superalloy [9], or high-entropy alloys [10,11,12]. Commercial ODS alloys are MA956 [13], MA957 [14], PM 2000, PM2010 [15,16], ODM alloys [17], and 1DK, 1DS [18]. Non-commercial, experimental, and advanced versions of ODS alloys include ODS Eurofer’97 [19], 9YWT [20], 12YWT [21], and 14YWT [22]. The excellent creep strength of the ODS steels is associated with an attractive interaction between dislocations and oxides. The stability of Y oxides as a strengthening agent is guaranteed up to 1300 °C [23]. The aim of the ongoing studies is to optimize Fe–Al–Cr–Y-O-based systems with high oxygen content (up to 1.5 wt. %) strengthened with yttria oxides (up to 5 vol.%). In the frame of these studies, the influence of metallic Y content and rolling temperature on the strength and creep properties were identified [24,25]. The new-generation ODS nanocomposite is called FeAlOY. The oxidation resistance of the FeAlOY, being better than that of Kanthal AF alloy, was analyzed in [26]. The major task ahead is to test long-term creep properties and proceed from the laboratory to industrial production of the FeAlOY. A comparison of the creep properties of our FeAlOY (rolled or swaged) with commercial creep-resistant alloys is shown in Figure 1. The Larson–Miller parameter *P* is a combined time–temperature parameter where *T* is the temperature in Kelvin, and *t* is the time in hours.

## 2. Materials and Methods

Initially, the development of the new, exclusively Fe–Al–O-based generation ODS alloys was aimed at the variations in the chemical composition and optimization of the processing, i.e., ball-milling process and hot-powder consolidation process, while keeping the alloy and processing as simple as possible. The optimum content of Al is close to 10 wt. %, which guarantees that intermetallic phases are not formed within the whole temperature range up to the melting temperature and the lattice remains always ferritic. In addition, Al significantly improves the oxidation resistance and decreases the alloy density. In the next steps of development, many trials with Cr, Mo, V, Ti, and Nb as alloying elements and also Al, Y, Zr, and La oxides have been performed to optimize the chemical composition of the FeAlOY with the aim to improve the cohesive strength of the grain boundaries and achieve coarse grains (at least 100 µm) after secondary recrystallization. Moreover, two methods of hot consolidation were studied—rolling and rotary swaging [27]. Also, the addition of up to 1.2 wt. % of metallic Y content to compensate for excess oxygen, number of rolling passes (2–6 passes, found optimum ~3 passes), and rolling temperature (860–960 °C) influence the strength and creep properties [24,25]. Top creep and oxidation resistance is achieved for an alloy of optimal chemical composition Fe–10Al–4Cr–4Y_2_O_3_ (wt.%). Four examples of well-performing alloys from the set of more than 150 trials are shown in this paper:

1. M16RS: Hot rotary swaged Fe–9Al–13Cr–4Mo–4Y_2_O_3_
2. M17: Hot rolled Fe–9Al–13Cr–4Mo–4Y_2_O_3_
3. A18RS: Hot rotary swaged Fe–10Al–3Y_2_O_3_–1Ti
4. Y9: Hot rolled Fe–10Al–4Cr–4Y_2_O_3_ (1% of metallic Y added)

**Note:** batches M—maraging steel as milling balls are used to eliminate C (the powder contains residues of about 0.9% Ni and 0.6% Co), batches A (no Cr added), batches Y (metallic Y added).

Two basic tensile specimen geometries of the secondary recrystallized coarse-grained FeAlOY are used in this study. Flat specimens with a gauge length of 25 mm and cross-section of 2.5 mm × 3.5 mm were cut by wire electro discharge machining (WEDM) from the rolled sheets, while cylindrical specimens with the gauge length of 25 mm and diameter of 6 mm with M10 threaded heads were produced from the swaged rods. The compression specimens were cylindrical with diameter of 6 mm and length 10 mm. Slow tensile tests at strain rate 10^−6^ s^−1^, creep compression tests, and some of the short-term tensile creep tests were performed using a creep test system Zwick/Roell KAPPA LA spring 50 kN equipped with vacuum furnace MAYTEC with operating temperatures up to 1400 °C (Zwick/Roell Testing Systems GmbH, Fürstenfeld, Austria). Most of the uniaxial creep tests were performed using in-house-built creep machines with a direct deadweight-loading system equipped by indirect measurement of the displacement on the loading train by laser sensor from Micro-Epsilon optoNCDT 1320 with 10 mm range.

The microscopic observations were performed using scanning electron microscope/microscopy (SEM) Tescan Lyra 3 XMU FEG/SEMxFIB (Tescan, Brno, Czech Republic) equipped with an X-Max80 EDS detector for X-ray microanalysis and a Symmetry S2 electron backscatter diffraction (EBSD) detector with an Aztec control system (Oxford Instruments, Abingdon, UK). The high-resolution transmission microcopy was performed using JEOL 2100F (JEOL Ltd., Akishima, Japan) at an acceleration voltage of 200 kV. The JEOL 2100F is operated in both TEM and STEM configurations with Gatan Tridem GIF (EFTEM, EELS) and Oxford X-MaxN TSR EDS detector.

## 3. Results

### 3.1. Powder Preparation and Processing of the FeAlOY

As inputs, 82% Fe powder, 10% Al powder, 4% small Cr granules, 3% Y_2_O_3_ powder, and 1% Y granules compensating for excess oxygen from oxidized powder surfaces and processing are used (wt.%). The following is the process in the ball mill:

(1) When the balls collide, small particles of powder are forged onto the balls and form larger and larger protrusions on their surface. When they grow sufficiently, they break off as large particles.
(2) The large particles are gradually broken into small ones and are forged onto the balls to grow the protrusions again.

**Figure 2:**
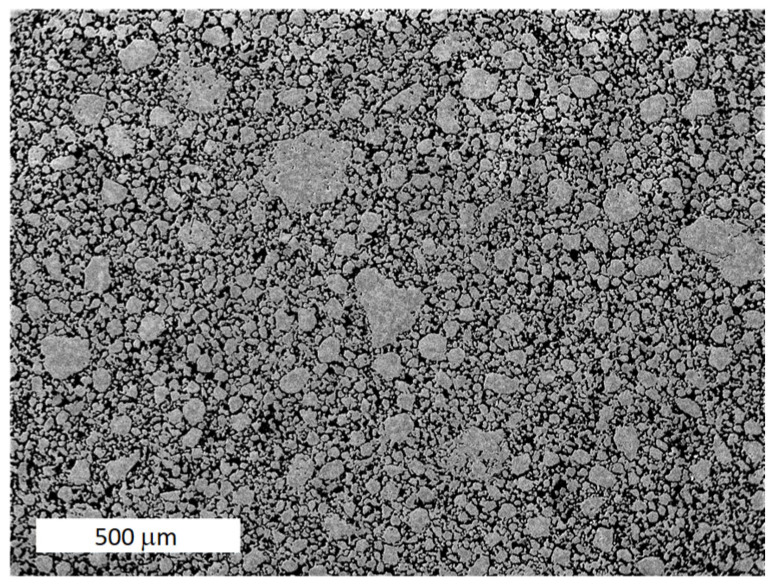
Compacted powder after mechanical alloying.

The process (1) and (2) are continually repeated. The resulting mechanically alloyed powder has a rather wide size distribution and is close to a spherical shape, which helps its very good compaction, as shown in Figure 2.

The conditions of the mechanical alloying are described in detail in [3]. The can with the cold compacted powder was evacuated for 10 min. by the rotary vacuum pump up to the pressure of 2 Pa and then sealed by welding.

### 3.2. Powder Consolidation and Microstructure

**Figure 3:**
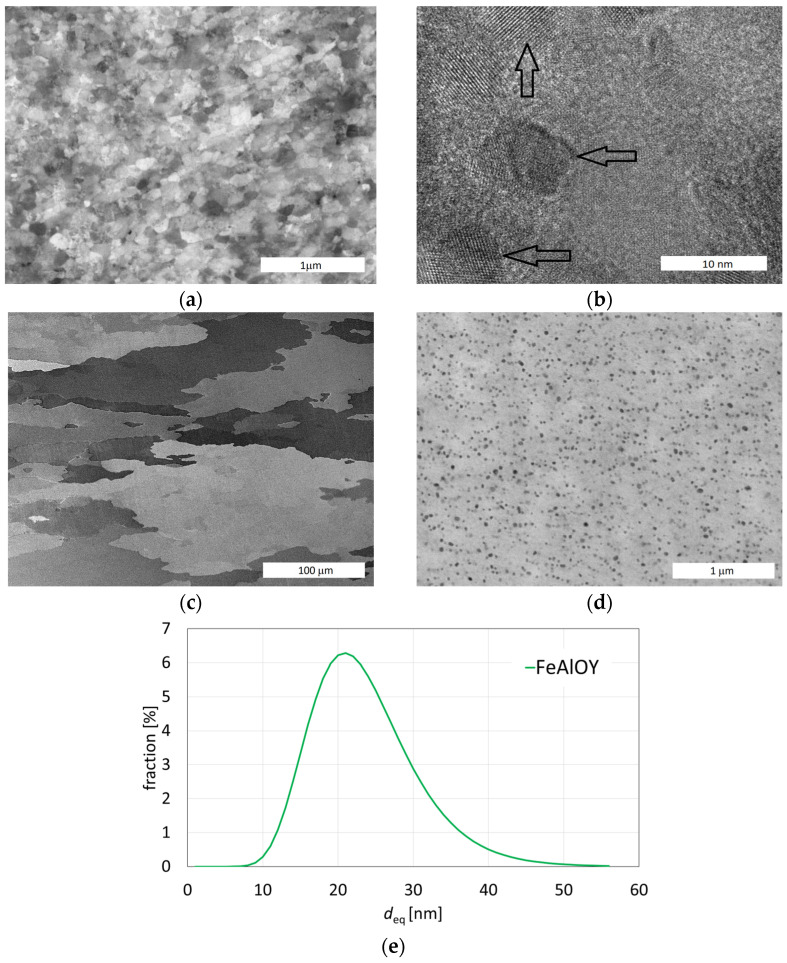
Microstructures of early experiments: (**a**) ultra-fine grains after hot rolling; (**b**) detail of Y_2_O_3_ oxides in the matrix marked by arrows; (**c**) secondary recrystallized microstructure; (**d**) dispersion of Y_2_O_3_ oxides in the matrix [28]; (**e**) distribution of the equivalent size of oxides.

ODS powder evacuated in a can is heat-consolidated by rolling or rotary swaging to the extent that the porosity is completely removed. After stripping off the can, a compact material is obtained with an ultrafine grain microstructure (typically 100 nm in size) reinforced by nanodispersion (typically 5 nm in size) of Y_2_O_3_. The presence of Y_2_O_3_ in the bulk of the FeAlOY was proven experimentally by both EDS and XRD methods [26]. The material is very brittle and practically unusable. Such microstructure is shown in Figure 3a, and the detail of Y_2_O_3_ oxides in the ferritic matrix is presented in Figure 3b, marked by arrows.

At 1200 °C, few grains grow rapidly at the expense of the ultrafine grains until the FeAlOY completely recrystallizes. Annealing at 1200 °C/2 h is generally sufficient for complete recrystallization. A recrystallization study that explains this process was published by Bártková et al. [29]. Typical microstructure after hot rolling and recrystallization is shown in Figure 3c, and the fine dispersion of oxides in the ferritic matrix is shown in Figure 3d. The distribution of the particle size is shown in Figure 3e. After the recrystallization, the FeAlOY becomes relatively ductile and easy to machine at room temperature and has excellent creep and oxidation resistance at high temperatures of up to 1300 °C.

### 3.3. Low Strain Rate Tensile Testing to Find Optimum Alloy

**Figure 4:**
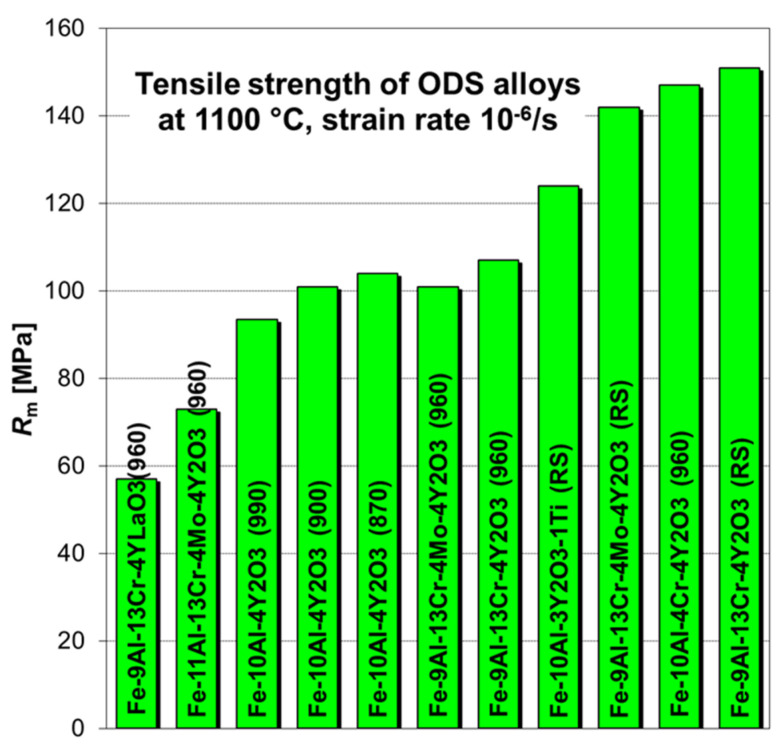
Comparison of tensile strength of ODS alloys at 1100 °C with various chemical compositions and consolidation temperature (RS—consolidated by rotary swaging).

A low strain rate tensile testing at 1100 °C and 10^−6^ s^−1^ was applied as a screening test to find optimum alloy composition and metallurgical-powder-processing conditions. Usually, 2–3 specimens of each batch produced are tested, and the strongest batch is then selected for creep testing, which is much more time consuming. The average values of tensile strength for some of the batches are shown in Figure 4. The last consolidation temperature of the rolled batches is presented in the parenthesis, and the rotary swaged batches are marked as RS.

### 3.4. Creep Testing

**Figure 5:**
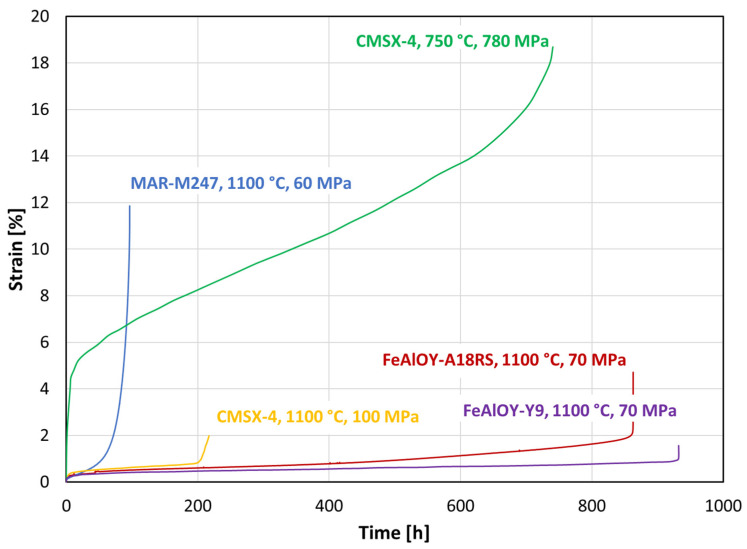
Comparison of two batches of FeAlOY with Ni-based polycrystalline superalloy MAR–M247 and single crystal CMSX-4 creep curves [28].

Creep testing is quite time- and energy-consuming, so only the most promising batches are selected for creep testing. A comparison of creep curves of FeAlOY–A18RS with Ni-based polycrystalline superalloy MAR–M247 and single crystal CMSX-4 is shown in Figure 5. Unlike the Ni-based polycrystalline alloys that spend most of their lifetime in the tertiary (accelerating) stage, the typical creep curve of FeAlOY has a very short primary stage, and most of the lifetime is spent during very slow secondary (stationary) creep, followed by a short tertiary stage. The creep curve of FeAlOY at 1100 °C resembles rather that of the Ni-based single crystal.

**Figure 6:**
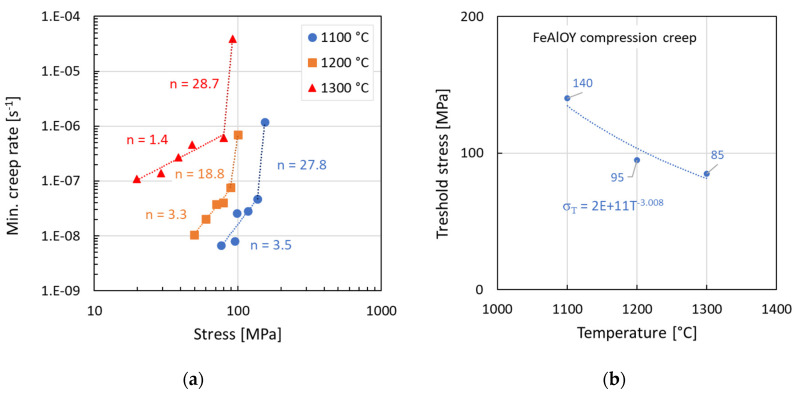
Compression creep test results of FeAlOY M16RS batch: (**a**) minimum creep rate vs. stress; (**b**) estimated threshold stress.

The compression creep testing allows for quick characterization of the creep resistance of the new material. The specimens are small and compact, so not too much material is needed for the testing. Figure 6 summarizes the creep behavior of the FeAlOY batch M16RS that was tested at three temperatures. The specimen was incrementally loaded, and after stabilization of the creep rate, the load was increased. The stress was calculated as the true stress, considering the constant volume of the cylindrical specimen. The data were fitted in two regions of applied stress by Norton power-law:

(1) $$\dot{\varepsilon }=A\sigma^{n},$$

where *A* is a material constant, and *n* is the stress exponent. One can note a sharp change in the stress exponent after reaching a certain level of stress in Figure 6a. This stress can be considered as the threshold stress, and its temperature dependence is shown in Figure 6b. The stress exponent *n* is notably decreasing with the increasing temperature, especially at 1300 °C.

The dependence of the creep rate on the applied stress σ at a given absolute temperature *T* was analyzed using the combination of power law and the Arrhenius equation:

(2) $$\dot{\varepsilon }=A_{0}\sigma^{n}\exp\left(-\frac{Q_{c}}{RT}\right),$$

where *A*_0_ is a material constant, *n* is the stress exponent, *Q*_c_ is the apparent activation energy of creep, and *R* is the universal gas constant. The values of *n* and *Q*_c_ can be found as partial derivatives of this equation:

(3) $$n={\left(\frac{\partial ln\dot{\varepsilon }}{\partial ln\sigma }\right)}_{T},$$

(4) $$Q_{c}=-{\left(\frac{\partial ln\dot{\varepsilon }}{\partial \frac{1}{RT}}\right)}_{\sigma }$$

**Figure 7:**
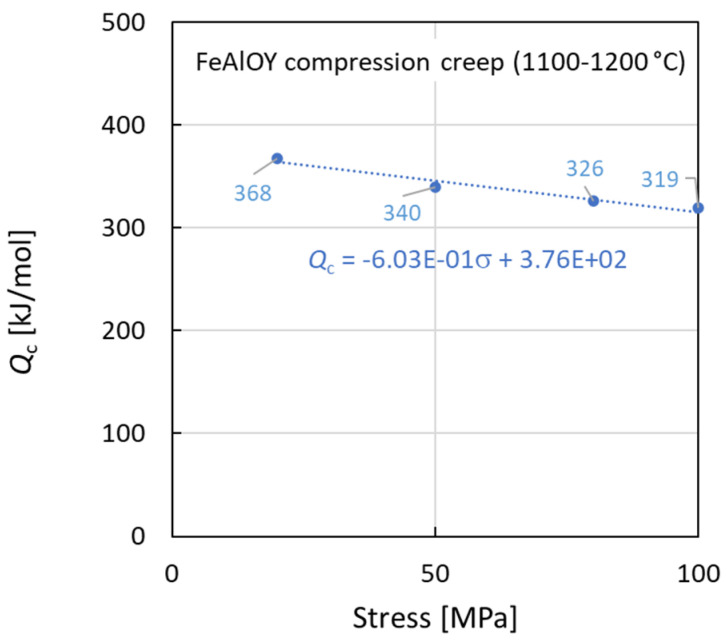
Activation energy of creep at *T* = 1100–1200 °C.

The creep activation energy calculated for stress 100 MPa and temperatures 1100–1200 °C is 319 kJ/mol. The activation energy slightly grows with decreasing stress to a value of 340 kJ/mol at 50 MPa as shown in Figure 7.

**Figure 8:**
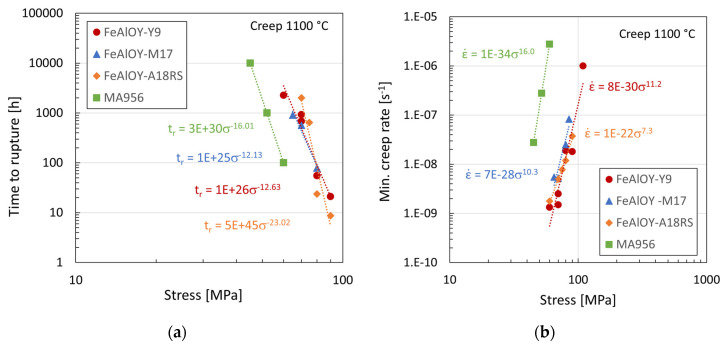
Tensile creep test results of FeAlOY: (**a**) time to rupture vs. stress; (**b**) creep rate vs. stress.

The tensile creep data are summarized and compared with the data of MA956 ODS alloy in Figure 8. All batches of FeAlOY outperform the MA956 both in time to rupture and minimum creep rate vs. stress. The stress for reaching approximately the same time to rupture is about 20 MPa higher for the FeAlOY, which is evident from Figure 8a. Figure 8b shows that the minimum creep rate at the same stress is about 2–3 orders lower for the FeAlOY, which is a significant improvement in the creep properties. The different batches of the FeAlOY behave very similarly; there are just small differences in the stress exponents between them.

**Figure 9:**
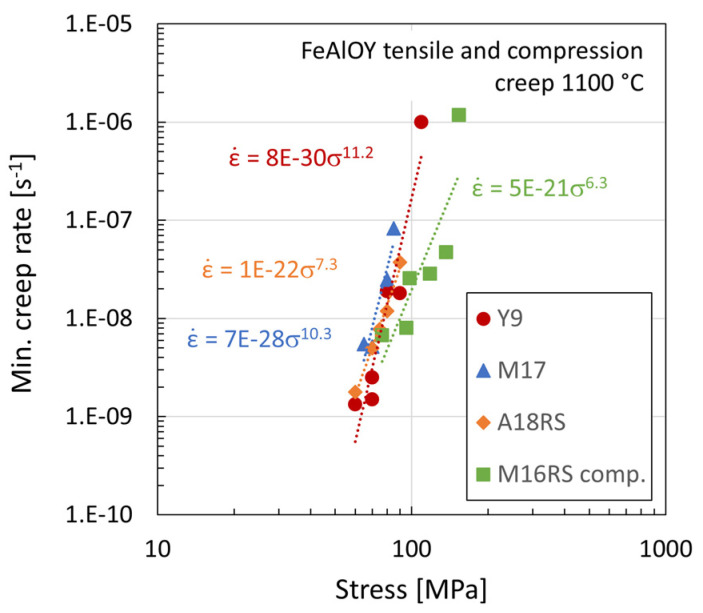
Creep rate vs. stress on various batches of FeAlOY.

A comparison of the creep data of all FeAlOY batches in tension and compression is shown in Figure 9. It is obvious that the compression data show slightly lower creep rates than tensile data, which is possible to explain by the different characteristics of the damage process in the specimens loaded by compression compared to tension. The decohesion of grain boundaries will be much lower or practically non-existent in the compression specimen. Therefore, especially at higher stress and creep rates, the difference is much more pronounced. The compression creep specimens did not have any signs of cracks and formed a barrel-like shape.

### 3.5. Fractographic and Microstructure Analyses of Ruptured Specimens

**Figure 10:**
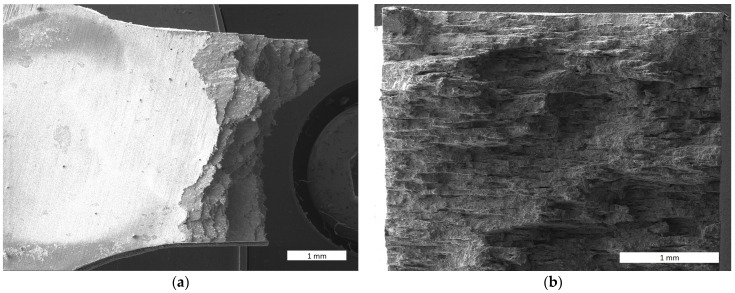
Fracture surface of the FeAlOY Y9 at 1100 °C, 10^−6^ s^−1^, *R*_m_ = 120 MPa: (**a**) top view; (**b**) view in the axial direction.

The observation of fractographs of the slow tensile test in Figure 10 reveals that the layers of pancake-like grains fracture at a minimal plastic deformation. The ductility of the specimen is only about 1%. The fracture looks macroscopically similar to that of layered composite materials. This underlays the conclusion that the cohesive strength of the grain boundaries is the factor limiting the strength of the FeAlOY in the temperature range 1000–1300 °C.

**Figure 11:**
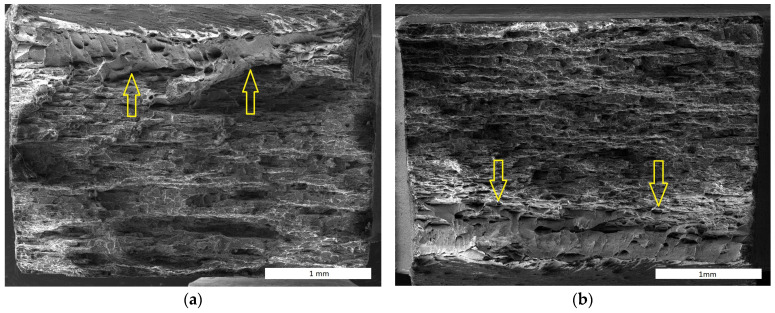
Creep fracture surface of the specimen Y9 tested at 1100 °C: (**a**) 60 MPa, *t*r = 2263 h; (**b**) 70 MPa, *t*r = 928 h; the arrows mark ductile fracture.

**Figure 12:**
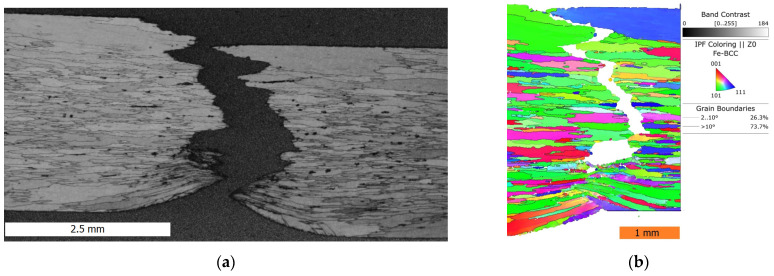
The vertical section along the symmetry axis of the fractured area of the Y9 specimen tested at 1100 °C and 60 MPa, *t*r = 2263 h: (**a**) backscattered electron image, (**b**) EBSD mapping.

The character of creep fracture shown in Figure 11 is similar to that of the tensile test, with only a notable higher plastic deformation in the part of the FeAlOY rupturing suddenly at the end of the test (marked by arrows). This part of the specimen close to one surface has the notable dimples characteristic of the ductile fracture. The character of the fracture can be better observed in Figure 12, which contains a vertical section along the symmetry axis of the fractured area. The fracture shows a prevailing intergranular decohesion character and intergranular cavity damage in the whole volume of the specimen, complemented by the ductile character of the final fracture in the necked area.

## 4. Discussion

The RS batches were initially notably stronger than batches consolidated by hot rolling, which was demonstrated in the creep study of a A18RS alloy [27]. The main reason was the shape and size of the recrystallized grains. While the “pancake-like” grains recrystallized after hot rolling were finer, the “needle-like” grains recrystallized after RS were much coarser, with a very high aspect ratio. As the powder processing and consolidation were more and more optimized, the difference in tensile strength after consolidation by hot RS and rolling was diminishing, which is visible in Figure 4.

The creep strength of the FeAlOY outperforms the MA956 ODS alloy by about 20 MPa to reach the same time to rupture or creep strain rate. From the results, it is clear that a higher density of nano-oxides may play a crucial role in the creep performance of the alloy. However, the chemical composition of the FeAlOY could also be more appropriate to increase the cohesive strengths of the grain boundaries.

The creep fracture of the FeAlOY at a temperature of 1100 °C is primarily driven by grain decohesion, so it has a mainly intergranular character with occasional transgranular damage of some grains. Partial ductile fracture characterized by high plastic deformation and necking of the grains near one of the specimen surfaces occurs once the cross-section is significantly reduced by the main fracture propagation.

It must be noted that the time to 1% of the creep strain is relevant to practical applications. There is still a need for creep testing at very low strain rates below 10^−9^ s^−1^ to fully understand the long-term creep behavior of the FeAlOY. The recent experiments clearly indicate a very small creep strain in the primary creep stage and very low creep rates in the secondary creep stage, which makes the FeAlOY (together with excellent oxidation resistance up to 1300 °C) very attractive to applications.

The literature data of creep activation energy of ODS alloys are quite rare at temperatures over 1000 °C, but comparable values of 348 kJ/mol were found for 0.35% yttria dispersed iron at 535–575 °C and 263 kJ/mol for pure iron [30]. Much higher values of *Q*_c_ are found for Ni-based superalloy MA754 962 kJ/mol for 950–1000 °C and 130–190 MPa [9]. In contrary, comparable values of *Q*_c_ were found for CrCoNi ODS alloy 335–367 kJ/mol for temperatures 700–900 °C and 60–200 MPa [12].

The hot consolidation of the FeAlOY seems to be the most complicated and expensive processing step. To achieve that, the FeAlOY becomes an attractive structural material, it is necessary to manage the hot consolidation of large cans filled with the FeAlOY powder in the most passable way. Just hot rolling of sheets and rods seems to be the most promising one and our future research will be concentrated also to this field.

## 5. Conclusions

Newly developed ODS alloys called FeAlOY are compared to currently known ODS alloys with 5–10× higher oxide content. Additional metallic Y is able to compensate for the oxygen in the powder (ensures precipitation of pure Y_2_O_3_), positively influence the secondary recrystallization (coarser grain microstructure), and significantly improve the strength at 1100–1300 °C. The overall performance of the FeAlOY can be summarized as follows:

- Long-term oxidation resistance up to 1300 °C.
- Long-term stability of strengthening nanodispersoid up to 1300 °C.
- High creep resistance at 1100–1300 °C exceeding the ODS alloy MA956.
- Over 100 MPa tensile and compression strength at 1100–1300 °C.
- Nearly no primary creep and low creep rate at 1100–1200 °C.
- Machinability in secondary recrystallized state comparable to carbon steels.
- Good hot formability in nanocrystalline state after hot consolidation.
- Production of bars up to 50 mm in diameter and sheets up to 100 mm in width by hot rolling is under development.

## Data Availability

The original contributions presented in the study are included in the article, further inquiries can be directed to the corresponding author.
